# Comparing the health of low income and less well educated groups in the United States and Canada

**DOI:** 10.1186/1478-7954-5-10

**Published:** 2007-10-16

**Authors:** Ken Eng, David Feeny

**Affiliations:** 1 Institute of Health Economics, Edmonton, AB, Canada; 2 Departments of Economics and Public Health Sciences, University of Alberta, Edmonton, Alberta, Canada; 3 Kaiser Permanente Northwest Centre for Health Research, Portland Oregon, USA; 4 Health Utilities Incorporated, Dundas, Ontario, Canada

## Abstract

**Background:**

A limited number of health status and health-related quality of life (HRQL) measures have been used for inter-country comparisons of population health. We compared the health of Canadians and Americans using a preference-based measure.

**Methods:**

The Joint Canada/United States Survey of Health (JCUSH) 2002–03 conducted a comprehensive cross-sectional telephone survey on the health of community-dwelling residents in Canada and the US (n = 8688). A preference-based measure, the Health Utilities Index Mark 3 (HUI3), was included in the JCUSH. Health status was analyzed for the entire population and white population only in both countries. Mean HUI3 overall scores were compared for both countries. A linear regression determinants of health model was estimated to account for differences in health between Canada and the US. Estimation with bootstraps was used to derive variance estimates that account for the survey's complex sampling design of clustering and stratification.

**Results:**

Income is associated with health in both countries. In the lowest income quintile, Canadians are healthier than Americans. At lower levels of education, again Canadians are healthier than Americans. Differences in health among subjects in the JCUSH are explained by age, gender, education, income, marital status, and country of residence.

**Conclusion:**

On average, population health in Canada and the US is similar. However, health disparities between Canadians and Americans exist at lower levels of education and income with Americans worse off. The results highlight the usefulness of continuous preference-based measures of population health such as the HUI3.

## Background

Canada and the United States are often compared in health studies, given the similarities between the two countries [1-3]. The Joint Canada/United States Survey of Health (JCUSH) 2002–03 compared the health status of Canadians and Americans by collecting the same information in the same manner for residents of both countries during the same time period [4-6]. The JCUSH included a preference-based measure of health-related quality of life, the Health Utilities Index Mark 3 (HUI3).

Limitations of previous studies comparing health in different countries include differences in instruments and methodologies. A comprehensive discussion of the issues involved in comparing population health across countries is provided elsewhere [7,8]. Evaluations of population health have usually only examined a single country [9]. In general, direct comparisons between countries with a continuous preference-based measure could not be made. The JCUSH overcomes these hurdles. The aim of our study was to compare the health status of Canadians and Americans based on data from the JCUSH using overall HUI3 scores as well as self-rated health. The study uses a linear multivariate regression determinants of health model to examine the differences in HUI3 scores between the two countries. Given the importance of race in the United States [10,11], additional analyses comparing the white population only in the US and Canada were undertaken. Furthermore, the health of Canadians and those in the US with and without health insurance are compared.

## Methods

### Study design

The JCUSH was conducted as a one-time telephone survey in both Canada and the US by Statistics Canada's regional offices between November 2002 and March 2003. The target population consisted of Canadian and American adults aged 18 and older residing in households. A proxy respondent was used if the respondent selected was unable to respond on his/her own behalf. Among the groups excluded from the target population were residents of institutions, full time members of the Canadian or American Armed Forces and residents of the Canadian or US territories.

The sample was stratified by Canadian province and by four US geographic regions (Northeast, Midwest, West and South) according to gender and three age groups (18–44, 45–64, and 65 and over). The sample was allocated proportionally within each stratum based on population sizes where households were selected based on a Random Digit Dialling (RDD) process.

### Measures

The HUI3 is a preference-based measure of HRQL that uses a multiplicative multi-attribute utility function that provides overall utility scores for HUI3 health states on the conventional dead = 0.00 to perfect health = 1.00 scale [12-14]. The HUI3 covers eight attributes of health status: vision, hearing, speech, ambulation, dexterity, emotion, cognition, and pain, with five or six levels per attribute. Overall HUI3 scores range from -0.36 (the all-worst HUI3 state) to 0.00 for dead to 1.00 for perfect health. Single-attribute utility scores range from 0.0 (lowest level, most impaired, such as deaf for hearing) to 1.0 for level 1 (no impairment).

Differences of 0.03 or more in overall HUI3 utility scores are regarded as clearly clinically important [15-17]. Differences of 0.01 may be important, especially in the context of population health [17]. For purposes of this paper with a focus on population health rather than clinical applications, clinically important differences will instead be referred to as quantitatively important differences.

Health-related quality of life was also assessed using the single-item self-rated health question: in general would you say your health is excellent, very good, good, fair, or poor?

### Statistical analyses

Statistical analyses were performed based on the entire sample and on the white population only from the JCUSH. HUI3 descriptive statistics (mean, median, minimum, maximum, standard deviation) were calculated and compared between the US and Canada. All of the analyses were conducted using sample weights to account for the target population size (not sample size of the JCUSH). The sample weight corresponds to the number of persons represented by the respondent with respect to the target population [18]. All analyses were performed on SAS version 9.1 (SAS Institute Inc., Cary North Carolina). Analyses were also conducted by health insurance status.

A linear regression determinants of health model with bootstraps was estimated to standardize the comparison due to slight differences in the age and gender distributions in the US and Canadian surveys. The survey's complex sampling design based on clustering and stratification requires that bootstraps are used to derive variance estimates [18]. Bootstraps involve the selection of random samples or replicates and calculate the variation from replicate to replicate. In each replicate, the survey weight for each record is recalculated. These weights are adjusted and post-stratified according to population estimates [18]. The entire process of selecting random samples, recalculating and post-stratifying weights for each stratum is repeated 1000 times to obtain variance estimates.

The determinants of health framework used here has been used in a number of previous studies [15,19-21]. The variables selected take into account both inter-country differences in health and confounders that have an impact on health.

Overall HUI3 scores were estimated as a function of a set of potential confounders. The list of confounders included age, gender, proxy report, education, marital status, Body Mass Index (BMI), income quintiles, health insurance and country of residence (see Table 1). Income quintiles in the JCUSH were defined according to the following. First, each respondent's household income was adjusted for household size by dividing the income by the square root of the number of persons residing in the household [6]. This methodology takes into account on the number of people living in the household [22]. Respondents were then ranked according to the adjusted household income and were assigned a quintile group such that the weighted count of each quintile group contained approximately one-fifth of the population reporting household income. Respondents with missing household income were excluded from the construction of quintiles and are reported separately [6].

**Table 1:** Description of variables in determinants of health regression model

**Variable Name**	**Definition**	**Comparator**
**Gender**	Female	Male
		
**Proxy**	Proxy response	Non proxy response
		
**Education**		
High School	High school educated	No high school
College/Technical College	College educated	No college
University	University educated	No university
		
**Marital Status**	Respondent's martial status is:	
Widow	Widow	Not widowed
Divorced	Divorced	Not divorced
Single	Single	Not single
		
**Purchasing power parity adjusted income quintile**		
Lowest income	$0–$17,499	Not $0–$17,499
Lower middle income	$17,500–$28,799	Not $17,500–$28,799
Higher middle income	$42,000–$62,353	Not $42,000–$62,353
Highest income	>$62,353	Not >$62,353
		
**Missing income**	Missing income data	No missing income data
		
**Body Mass Index (BMI)**		
BMI0 (underweight)	BMI <18.5	BMI not <18.5
BMI1 (overweight)	BMI 25–29.9	BMI not 25–29.9
BMI2 (obese)	BMI > = 30	BMI not > = 30
		
**Age Categories**		
Age	45–64	Not 45–64
Age1	65–74	Not 65–74
Age2	75+	Not 75+
		
**Uninsured Americans**	Americans with no health insurance	Americans with health insurance
		
**Country**	US	Canada

Table 1 provides a summary and description of the variables in the regression model. Individual dummy variables were constructed for high school, technical college and university as indicators for education. Marital status had separate dummy variables for those who were widowed, divorced and single. Income had separate variables according to lowest, lower middle, higher middle and highest income quintiles. Income quintiles were redefined for both countries in a pooled sample and adjusted for purchasing power parity in Canadian dollars to account for price differences between countries. The income distribution was defined over the joint distribution to derive the quintiles. Approximately 20% of income data are missing. Those for whom income data were missing were treated as a separate group in the regression analyses [23,24]. BMI was categorized according to the classification underweight (<18.5), normal (18.5 – 24.9) overweight (25.0 – 29.9) and obese (≥ 30). Age was based on age groups (45–64, 65–74, 75+). In addition, separate dummy variables were used in the model to identify country and Americans without health care insurance. Finally, because HUI3 data are skewed, additional analyses based on the natural logarithm transformation of HUI3 scores were also conducted.

## Results

A total of 8,688 people responded to the JCUSH. Response rates were 66% and 50% in Canada and the US; 3,505 (40%) of respondents were from Canada and 5,183 (60%) from the United States. For the "whites only" population, the total sample size is 6,716 with 2,890 Canadians and 3,826 Americans. The "whites only" population, therefore, represents 82% of the entire sample in Canada and 74% in the US. The total number of proxy respondents was small. Only 1.5% of responses were completed by a proxy in Canada and 3.3% in the US.

The mean HUI3 score for Canadians (mean HUI3 = 0.88, sd = 0.20) was slightly higher than the one for Americans (mean HUI3 = 0.87, sd = 0.21). The difference was statistically significant at 5% (p-value = 0.00) Canadians in the JCUSH were slightly younger with a higher proportion of males (mean age 45.11 and 49% males) compared to the US (mean age 45.35 and 48% males). Results for the "whites only" group were similar.

For both countries mean HUI3 overall scores were similar for each category of self-rated health (Table 2). Quantitatively important and statistically significant differences at 5% in HUI3 scores were found for respondents in poor health (difference 0.07, p-value = 0.01) between US and Canada. Overall HUI3 scores for "whites only" exhibit a similar pattern in which quantitatively important differences and statistical significance were found between Americans and Canadians in poor health (difference 0.09, p-value = 0.00).

**Table 2:** Self-rated health status and overall HUI3 utility scores

	**Entire Sample**			**Whites only**		
**Self-rated health**	**Canada **mean HUI3 (% of people)	**US **mean HUI3 (% of people)	**Mean difference **(p-value)	**Canada **mean HUI3 (% of people)	**US **mean HUI3 (% of people)	**Mean difference **(p-value)
**Excellent**	0.96 (24%)	0.95 (27%)	0.01 (0.70)	0.96 (25%)	0.95 (28%)	0.01 (0.71)
**Very good**	0.92 (37%)	0.92 (33%)	0.00 (0.68)	0.92 (38%)	0.92 (36%)	0.00 (0.81)
**Good**	0.87 (28%)	0.86 (26%)	0.01 (0.11)	0.87 (26%)	0.85 (25%)	0.02 (0.14)
**Fair**	0.69 (8%)	0.71 (10%)	-0.02 (0.68)	0.70 (8%)	0.70 (8%)	0.00 (0.63)
**Poor**	0.42 (3%)	0.35 (4%)	0.07 (0.01)	0.42 (3%)	0.33 (3%)	0.09 (0.00)

Mean difference is Canadian mean HUI3 score less US mean HUI3 scores. P-values were based on mean differences for each group.

Overall HUI3 scores (Table 3) were examined for each household income quintile. The lowest scores were found in the lowest income quintile in both countries (Canada = 0.81, US = 0.77); scores were highest for the highest income quintile (Canada = 0.93, US = 0.93) based on results for the entire sample. Quantitatively important differences in scores and statistical significance between the countries were found only for the 1^st ^(lowest) income quintile (difference 0.04, p-value = 0.04). Similar results were found for "whites only" for which quantitatively important differences were also found only for the 1^st ^income quintile (difference 0.05).

**Table 3:** Overall HUI3 scores by household purchasing power parity adjusted income

	**Entire sample**			**Whites only**		
**Income quintile**	**Canada **mean HUI3	**US **mean HUI3	**Mean difference **(p-value)	**Canada **mean HUI3	**US **mean HUI3	**Mean difference **(p-value)
1^st ^(lowest)	0.81	0.77	0.04 (0.04)	0.79	0.74	0.05 (0.07)
2^nd^	0.86	0.85	0.01 (0.80)	0.85	0.83	0.02 (0.34)
3^rd^	0.90	0.89	0.01 (0.31)	0.90	0.87	0.03 (0.24)
4^th^	0.92	0.91	0.01 (0.43)	0.92	0.91	0.01 (0.48)
5^th ^(highest)	0.93	0.93	0.00 (0.95)	0.93	0.93	0.00 (0.98)

Mean difference is Canadian mean HUI3 score less US mean HUI3 scores. P-values were based on mean differences for each group.

HUI3 scores for the US and Canada were similar for the top three levels of education (high school, Canada = 0.89, US = 0.86; technical school/college Canada = 0.89, US = 0.87; bachelor degree Canada = 0.92, US = 0.92) (Table 4). In both countries the lowest scores were for respondents with the least education (less than high school Canada = 0.81, U.S = 0.74). Quantitatively important and statistically significant differences in health were found between Canadian and American respondents for the bottom two levels of education (less than high school = 0.07, p-value = 0.00; high school = 0.03, p-value = 0.03). The pattern of scores for "whites only" was similar. Quantitatively important differences between white Canadians and Americans were found for those with less than high school (0.07), high school (0.03) and technical/college education (0.03). Statistical significance at 5% was found for those with less than high school (p-value = 0.00) and technical/college education (p-value = 0.03).

**Table 4:** Overall HUI3 scores by education

	**Entire sample**			**Whites only**		
**Education**	**Canada **mean HUI3	**US **mean HUI3	**Mean difference **(p-value)	**Canada **mean HUI3	**US **mean HUI3	**Mean difference **(p-value)
Less than high school	0.81	0.74	0.07 (0.00)	0.81	0.74	0.07 (0.00)
High school	0.89	0.86	0.03 (0.03)	0.89	0.86	0.03 (0.07)
Technical college	0.89	0.87	0.02 (0.13)	0.89	0.86	0.03 (0.03)
University degree	0.92	0.92	0.00 (0.91)	0.92	0.92	0.00 (0.80)

Mean difference is Canadian mean HUI3 score less US mean HUI3 scores. P-values were based on mean differences for each group.

The regression models are displayed in Tables 5 (entire sample) and 6 ("whites only"). The results from Table 5 suggest that respondents found to be healthier were more educated, younger, married, male, and had higher income. The country of residence dummy variable was statistically significant, indicating that controlling for confounders, Canadians were healthier than Americans. Most variables in the model were statistically significant (at 5%) and quantitatively important except for gender, being single, being in the higher middle-income quintile, being overweight (BMI 25–29.9) and being an uninsured American. Similar results (not shown) were also found for regressions that excluded respondents for whom income data were missing. Results for "whites only" (Table 6) were similar.

**Table 5:** Regression entire sample. HUI3 = f(gender, proxy reporting, education, martial status, income, missing income, BMI, age, country)

	**Estimated Coefficient**	**Standard Error**	**T-Statistic**	**P-Value**
**Constant**	0.89	0.01	65.32	0.00
**Gender (Female)**	-0.01	0.01	-1.90	0.06
**Proxy report**	-0.13	0.03	-5.02	0.00
**High school**	0.07	0.01	5.40	0.00
**College**	0.07	0.01	4.96	0.00
**University**	0.10	0.01	7.58	0.00
**Widow**	-0.03	0.02	-2.00	0.05
**Divorce**	-0.04	0.01	-4.27	0.00
**Single**	-0.01	0.01	-1.32	0.19
**Lowest income**	-0.08	0.01	-6.48	0.00
**Lower middle income**	-0.02	0.01	-2.11	0.03
**Higher middle income**	0.01	0.01	1.60	0.11
**Highest income**	0.02	0.01	3.04	0.00
**Missing Income**	-0.01	0.01	-1.38	0.17
**BMI0 (underweight)**	-0.05	0.02	-2.35	0.02
**BMI1 (overweight)**	-0.01	0.01	-1.54	0.12
**BMI2 (obese)**	-0.07	0.01	-7.19	0.00
**Age (45–64)**	-0.06	0.01	-7.85	0.00
**Age1 (65–74)**	-0.06	0.01	-5.56	0.00
**Age2 (75+)**	-0.13	0.02	-8.17	0.00
**US no insurance**	0.00	0.01	-0.35	0.73
**Country (USA)**	-0.02	0.01	-2.84	0.00

Adjusted R^2^

0.15. Omitted categories: middle income quintile ($28,800–$41,999); BMI 18.5–24.9 (normal weight); Age 18–44; less than high school; male; married

**Table 6:** Regression for whites only. HUI3 = f(gender, proxy reporting, education, martial status, income, missing income, BMI, age, US without health insurance, country)

	**Estimated Coefficient**	**Standard Error**	**T-Statistic**	**P-Value**
**Constant**	0.89	0.02	57.31	0.00
**Gender (Female)**	-0.01	0.01	-1.84	0.07
**Proxy report**	-0.14	0.03	-4.52	0.00
**High school**	0.07	0.01	4.84	0.00
**College**	0.06	0.02	3.73	0.00
**University**	0.10	0.01	6.54	0.00
**Widow**	-0.03	0.01	-2.12	0.03
**Divorce**	-0.05	0.01	-4.15	0.00
**Single**	0.00	0.01	-0.56	0.57
**Lowest income**	-0.07	0.01	-5.29	0.00
**Lower middle income**	-0.04	0.01	-3.12	0.00
**Higher middle income**	0.01	0.01	1.14	0.25
**Highest income**	0.02	0.01	2.81	0.01
**Missing Income**	-0.01	0.01	-0.67	0.51
**BMI0 (underweight)**	-0.03	0.02	-1.33	0.18
**BMI1 (overweight)**	0.00	0.01	-0.15	0.88
**BMI2 (obese)**	-0.06	0.01	-6.02	0.00
**Age (45–64)**	-0.05	0.01	-7.42	0.00
**Age1 (65–74)**	-0.05	0.01	-5.19	0.00
**Age2 (75+)**	-0.12	0.02	-7.20	0.00
**US no insurance**	-0.02	0.01	-1.16	0.25
**Country (USA)**	-0.02	0.01	-2.92	0.00

Adjusted R^2^0.14

Omitted categories: middle income quintile ($28,800–$41,999); BMI 18.5–24.9 (normal weight); Age 18–44; less than high school; male; married

Regression models for the entire sample and for "whites only" were also conducted taking the natural log of HUI3. Results (not shown) were similar to those reported in Tables 5 and 6.

## Discussion

A comprehensive cross-country comparison of health status was made between Canada and the United States using a preference-based measure. Results are based on a survey that employed the same instruments and method at the same time in both countries.

Using alternative measures of health, Sanmartin *et al*. [25] report results from the JCUSH similar to those reported here. Sanmartin *et al*. report that the majority of respondents in both countries were in good, very good or excellent health. Sanmartin *et al*. found a higher proportion of US households in lower income groups with poor or fair health compared to Canadian households in the same income groups. Results from this study corroborate the results reported by Sanmartin *et al*. using a different measure of health; this study found lower mean HUI3 scores for Americans in lower income groups compared to their Canadian counterparts. Like this study, Sanmartin *et al*. did not find significant health differences between Canadians and Americans in higher income quintiles.

Using the JCUSH, Lasser *et al*. [23] focus on comparing low-income, racial and immigrant groups in the two countries. Lasser *et al*. report HUI3 scores for those in the lowest quartile at 0.760 in the United States and 0.786 in Canada. Lasser *et al*. found differences in health on the basis of race, income and immigrant status in both countries. However, racial disparities in health, although present in both countries, were more extreme in the United States. The analyses reported in this study are more comprehensive, relying on both the entire sample and the white only population from the JCUSH.

Armstrong *et al*. [26] also make use of the JCUSH. They rely mainly on self-rated health to compare the two countries. They also highlight disparities for recent immigrants, those with low levels of education, and those with low incomes. Like Evans and Roos [1], Manuel and Mao [3], Kunitz *et al*. [11], Lasser *et al*. [23] and Ross *et al*. [27], Armstrong *et al*. [26] suggest that key differences between the US and Canada include access to healthcare and the degree of social and economic inequality.

It is also important to compare our results to estimates of mean HUI3 scores for the US and Canada from earlier studies. Mean HUI3 overall scores from the entire sample in this study (0.88) are slightly lower than those reported by Maddigan *et al*. (0.90) [21]. The sample from Maddigan *et al*. comprised of Canadian respondents from the 1996–1997 Canadian National Population Health Survey (NPHS) Cycle 2. Response rate (82.6%) and sample size (66,093) in the NPHS were substantially higher than those in the JCUSH (66%, 8,688).

Similar to findings reported by Hopman *et al*. Canadians are slightly healthier than Americans [28]. Hopman *et al*. used the Medical Outcomes Study 36-item Short Form (SF-36) to evaluate HRQL. Canadian norms were higher than US norms for every SF-36 domain and both summary scores. The magnitude of the differences between Canada and the US were small. Similarly, in this study differences in HUI3 overall scores between the two countries were also small.

Mean HUI3 overall scores for Americans (0.87) in this study are noticeably higher than those reported by Luo *et al*. (0.81) [29]. Both studies had similar target populations of respondents 18 years or older residing in the community in the US. Response rates in this study (50%) were lower than in Luo *et al*. (59%). Sample size in the latter survey was lower (4048) compared to this study (5183). Both studies shared similar characteristics with more females (52%) and a mean age of 45.

As noted by Luo *et al*., the difference in the mode of administration is probably the most important factor in accounting for the difference in results between the two studies. HUI3 data in the survey reported on by Luo *et al*. were collected using a self-complete paper-and-pencil questionnaire. In contrast the JCUSH collected HUI3 data via telephone using an interviewer-administered questionnaire. A comparison of results from the Ontario Health Survey for two HUI3 attributes, pain and emotion, collected using both paper-and-pencil self complete and in-person interviewer administration reveals that the burden of morbidity reported on the self-complete questionnaire in general exceeded the burden reported on the interviewer- administrated questionnaire [30]. It is plausible that the mode of administration could account for most or all of the observed difference in mean scores between those reported by Luo *et al*. and those based on the JCUSH.

Limitations of the JCUSH include the somewhat modest response rates, 66% (Canada) and 50% (US), and that the response rate was higher in one country than the other. It is possible that non-response bias could affect the US-Canada comparison. However, if those with lower health status were less likely to respond than healthier respondents, then the JCUSH could have understated the difference between the two countries. A variable for health insurance was not included in the model due to collinearity with country. To account for this we included a variable for uninsured Americans. However, this was not found to be quantitatively important or statistically significant.

Additionally, the results reported here are based on the use of the HUI3 scoring function, based on preference scores obtained from a random sample of the Canadian population, to value health status observed both in Canada and the United States. The use of the same scoring function for both countries enhances the comparability of the results. It is possible that residents of the US might value health states differently than do residents of Canada. We are unaware of any direct evidence on this issue. However, it is important to note that the parameter values of HUI3 scoring functions estimated based on preference scores from the general population in France [31], Spain [32], and the Netherlands [33] are very similar to the parameter values of the function based on preferences scores obtained in Canada [12]. One might infer on the basis of this evidence that major differences in the preferences for health states in the US and Canada are unlikely. Nonetheless, this potential limitation should be kept in mind in interpreting the results reported here.

The results of this study indicate that on average health-related quality of life in Canada and the United States is very similar, with slightly higher mean scores in Canada. However, there is a substantial difference in health-related quality of life between the two countries for those with low income or low educational attainment. Canadian mortality also compares favourably to mortality in the US [3,11,34]. Estimates of life expectancy and the infant mortality rate from the Organisation for Economic Co-operation and Development (2006) further document the differences in mortality [34]. For 2002 life expectancy at birth for females, males, and the total population in Canada was 82.1, 77.2, and 79.7 years; corresponding results for the US are 79.9, 74.5, and 77.2 years. Similarly the infant mortality rate in Canada was 5.4 per 1,000 while the rate in the US was 7.0. Estimates of Health Adjusted Life Expectancy (HALE) for Canada and the US highlight the difference in morbidity and mortality between the countries [35]. Canadian HALE estimates for 2002 for males, females, and the total population was 70.1, 74.0, and 72.0. In the US those estimates were 67.2, 71.3, and 69.3.

## Conclusion

This study found that Canadians at low levels of education or income were substantially healthier than their US counterparts. HUI3 scores were higher for higher income categories in both countries. This relationship between health and income has been well documented [36,37]. Others have reported that household income is a strong predictor of health status [38]. Important differences in health between the two countries were found at less than high school and high school education. This is consistent with previous work in which educational attainment was positively associated with health [39,40]. Both quantitative importance and statistical significance were found for income and education in this study.

The strength of the JCUSH was the application of identical methodology and instruments. The findings in this study reveal health disparities between Canadians and Americans at lower levels of education and income, with Americans worse off. Differences between the two countries in social and economic inequality as well as in access to healthcare may account for the observed differences in the health of those with lower levels of education and/or income [41]. The social safety net that Canada provides compared to the US seems to have an impact on health for those with less education and income. The results highlight the usefulness of a continuous preference-based measure, in this case the HUI3, for making comparisons across time and space, in this case the US and Canada.

## Competing interests

It should be noted that David Feeny has a proprietary interest in Health Utilities Incorporated, Dundas, Ontario, Canada. HUInc. distributes copyrighted Health Utilities Index (HUI) materials and provides methodological advice on the use of HUI. Ken Eng has no conflict of interest to declare.

## Authors' contributions

DF conceived of the study and designed the analyses. KE participated in the design of the analyses and conducted the analyses. Both authors participated in drafting the manuscript.
